# Protective Effects of Green Tea Extract against Hepatic Tissue Injury in Streptozotocin-Induced Diabetic Rats

**DOI:** 10.1155/2012/740671

**Published:** 2012-02-27

**Authors:** Ali Akbar Abolfathi, Daryoush Mohajeri, Ali Rezaie, Mehrdad Nazeri

**Affiliations:** ^1^Department of Biological Science, Ahar Branch, Islamic Azad University, Ahar, Iran; ^2^Department of Pathobiology, Tabriz Branch, Islamic Azad University, Tabriz, Iran; ^3^Department of Clinical Science, Tabriz Branch, Islamic Azad University, Tabriz, Iran; ^4^Young Researchers Club, Tabriz Branch, Islamic Azad University, Tabriz, Iran

## Abstract

Although diabetic hepatopathy is potentially less common, it may be appropriate for addition to the list of target organ conditions related to diabetes. This study was designed to evaluate the hepatoprotective properties of green tea extract (GTE) in STZ-induced diabetes in rats. Wistar rats were made diabetic through single injection of STZ (75 mg/kg i.p.). The rats were randomly divided into four groups of 10 animals each: Group 1, healthy control; Group 2, nondiabetics treated with GTE administered orally (1.5%, w/v); Group 3, diabetics; Group 4, diabetics treated with GTE (1.5%, w/v) for 8 weeks. Serum biomarkers were assessed to determine hepatic injury. Malondialdehyde (MDA) and reduced glutathione (GSH) contents were measured to assess free radical activity in the liver tissue. Hepatic antioxidant activities of glutathione peroxidase (GSH-Px), superoxide dismutase (SOD), and catalase (CAT) were also determined. The biochemical findings were matched with histopathological verifications. Liver MDA content and serum levels of ALT, AST, ALP, and bilirubin in Group 3 significantly increased compared to Group 1 (*P* < 0.05) and significantly decreased in Group 4 compared to Group 3 (*P* < 0.05). Serum albumin level and GSH, SOD, CAT, and GSH-Px contents of the liver in Group 3 were significantly decreased compared to Group 1 (*P* < 0.05) and were significantly increased in Group 4 compared to Group 3 (*P* < 0.05). Histopathologically, the changes were in the same direction with biochemical findings. This study proved the hepatoprotective activity of GTE in experimentally induced diabetic rats.

## 1. Introduction

Type 1 and type 2 diabetes mellitus (T2DM) are influenced by different genetic factors, but individuals with either of them are prone to developing complications including nephropathy, retinopathy, peripheral neuropathy, and hypertension [1, 2]. Diabetes, by most estimates, is now the most common cause of liver disease in the USA and liver disease is an important cause of death in type 2 diabetes [3]. Thus, patients with diabetes have a high prevalence of liver disease and patients with liver disease have a high prevalence of diabetes. Virtually the entire spectrum of liver disease is seen in patients with type 2 diabetes. This includes abnormal liver enzymes, nonalcoholic fatty liver disease (NAFLD), cirrhosis, hepatocellular carcinoma, and acute liver failure [4]. Chronic mild elevations of transaminases are frequently found in type 2 diabetic patients. However, when the liver fails, there is no equivalent form of management, such as hemodialysis or retinal photo-coagulation. Thus, although diabetic hepatopathy is potentially less common, it may be appropriate for addition to the list of target organ conditions related to diabetes, such as glomerulopathy, retinopathy, and neuropathy. However, annual screening for liver disease might be accomplished by means of a simple biochemical analyte such as alanine aminotransferase [5].

Oxidative stress is currently suggested as a mechanism underlying diabetes and diabetic complications [6]. Free radicals are continually produced in the body as the result of normal metabolic processes and interaction with environmental stimuli. Under physiological conditions, a wide range of antioxidant defences protects against the adverse effects of free radical production in vivo [6]. Oxidative stress results from an imbalance between radical-generating and radical-scavenging systems, that is, increased free radical production or reduced activity of antioxidant defences or both these phenomena. In diabetes, protein glycation and glucose autoxidation may generate free radicals, which in turn catalyse lipid peroxidation [7, 8]. Moreover, disturbances of antioxidant defence systems in diabetes were shown: alteration in antioxidant enzymes [9], impaired glutathione metabolism [10], and decreased ascorbic acid levels [11, 12]. However, an increased oxidative stress in vivo has never been clearly demonstrated. Several studies in humans and animal models, using thiobarbituric acid reactive substances (TBARS) assay [13–18], have shown increased lipid peroxidation in membranes and lipoproteins in the diabetic state. However, Lipid peroxidation and antioxidant status of hepatic tissue were studied by Feillet-Coudray et al. in experimental diabetes [19].

Antidiabetic agents have generally been shown to decrease the levels of serum biomarker of hepatic injury [20], but these agents can produce serious side effects [21]. Natural remedies from medicinal plants are considered to be effective and safe alternative treatment for hyperglycemia and liver toxicity. There is a growing interest in herbal remedies because of their effectiveness, minimal side effects in clinical experience, and relatively low cost. Herbal drugs or their extracts are prescribed widely, even when their biological active compounds are unknown [22]. For example, beneficial effect of freshly prepared aqueous extracts of *Psidium guajava*, *Momordica charantia, *and *Coccinia indica* leaves and their combination in STZ-induced diabetes rats has been proved by Rafiq et al. [23]. Similarly, antidiabetic activity of hydroethanolic extracts of *Nymphaea stellata* flowers has been documented in alloxan-induced diabetic rats by Rajagopal and Sasikala [24]. Therefore, studies with plant extracts are useful to know their efficacy and mechanism of action and safety.

Green tea (leaves of *Camellia sinensis*, Theaceae) is a popular beverage in East Asia and also used as a herbal remedy in Europe and North America. An increased consumption of green tea may reduce the risk of liver disease. Population-based clinical studies have shown that men who drink more than 10 cups of green tea per day are less likely to develop disorders of the liver. Green tea also seems to protect the liver from the damaging effects of toxic substances such as alcohol [25]. Green tea is considered to be anti inflammatory, antioxidative, antimutagenic, and anticarcinogenic [26, 27], and can prevent cardiac disorders. Epidemiologically, it has been suggested that green tea consumption prevents type 2 diabetes. [28, 29]. Tsuneki et al. demonstrated that green tea produces an antihyperglycemic effect in STZ diabetic mice [30]. Crespy and Williamson [31] reported that green tea extract (GTE) displays antioxidants and free radicals scavenger properties.

Considering the antihyperglycemic properties and antioxidant activities of green tea, this study was designed to evaluate the hepatoprotective effects of green tea extract in streptozotocin-induced diabetes of rats. In this context, green tea can rightly be mentioned as a plant of considerable interest.

## 2. Materials and Methods

### 2.1. Chemicals

Streptozotocin was from Sigma (St. Louis, MO, USA). All other chemicals used were of analytical grade. All chemicals used in this study were of analytical grade and obtained from Nanjing Jiancheng Bioengineering Institute, Nanjing, China.

### 2.2. Green Tea Extract (GTE)

The green tea extract (GTE) was made according to Maity et al. [32], by soaking 15 g of instant green tea powder in 1 L of boiling distilled water for 5 min. The solution was filtered to make 1.5% GTE. This solution was provided to rats as their sole source of drinking water.

### 2.3. Induction of Diabetes Mellitus

Diabetes was induced by intravenous injection of streptozotocin (Sigma, St. Louis, Mo, USA) into the tail vein at a dose of 65 mg/kg body weight. STZ was extemporaneously dissolved in 0.1 M cold sodium citrate buffer, pH 4.5. After 18 h, animals with fasting blood glucose levels greater than 16.5 mmol/L were considered diabetic and then included in this study [33]. Fasting blood glucose was estimated by using one touch glucometer (Accu-Chek sensor) of Roche Diagnostics, Germany.

### 2.4. Animal Treatment

This experimental study was carried out in Islamic Azad University Research Center and all procedures and works on animals were conducted under Animal Rights Monitoring Committee of the Islamic Azad University Research Center.

Forty healthy male Wistar rats (about 180–200 g body weight) were purchased from Animal House, Islamic Azad University. All animals were conditioned at room temperature at a natural photoperiod for 1 week before experiment execution. A commercial balanced diet and tap water ad libitum were provided. The duration of experiment was 8 weeks. The rats were randomly divided into 4 groups (10 rats each) as follows: Group 1, healthy control rats received distilled water as sole drinking source; Group 2 non-diabetic rats GTE (1.5%, w/v) was given in drinking water; Group 3, diabetic and no treatment was given of beginning of experiment; Group 4, diabetic rats were treated with were treated with GTE (1.5%, w/v) as sole drinking source.

### 2.5. Biochemical Factors Evaluation

At the end of experiment, blood samples were collected from the retro-orbital plexus and the sera prepared through centrifuging at 2500 ×g for 15 minutes at 30°C. Serum biomarkers of liver function including alanine aminotransferase (ALT), aspartate aminotransferase (AST) [34], alkaline phosphatase (ALP) [35], albumin [36], and total bilirubin [37] were measured using commercially available kits. Aminotransferases (ALT and AST) were measured to determine the concentration of intracellular hepatic enzymes that have leaked into the circulation and serve as a marker of hepatocyte injury. ALP and bilirubin were measured to assess biliary function. Albumin was measured to reflect liver synthetic function.

### 2.6. Measurement of Antioxidant Activity

The rat's liver was removed immediately and washed in normal saline and homogenate 10% prepared in 1.15% w/v of potassium chloride. The homogenate was centrifuged in 7000 ×g for 10 minutes at 4°C and supernatant was used for measurement of oxidative stress by estimation of reduced glutathione (GSH) and determination of malondialdehyde (MDA) as well as antioxidant enzymes (AOEs) such as superoxide dismutase (SOD), catalase (CAT), and glutathione peroxidase (GSH-Px). GSH, MDA, SOD, CAT, and GSH-Px were measured by using commercially available kits according to the manufacturer's protocol (Nanjing Jiancheng Bioengineering Institute, Nanjing, China).

Reduced glutathione (GSH) content was determined according to Sedlak and Lindsay [38]. GSH reacts with 5,5′-dithiobis-2-nitrobenzoic acid, and the absorbance spectra of the product have a maximum absorbance at 410 nm. The results were expressed as *μ*mol/g wt. Liver homogenate MDA levels were expressed as nmol MDA per mg protein and antioxidant activity was expressed as arbitrary units per mg protein. Degree of lipid peroxidation in liver tissue homogenates was determined in terms of thiobarbituric acid reactive substances (TBARSs) formation by following the protocol of Esterbauer and Cheeseman [39]. SOD activity was measured by Nishikimi et al. method [40] and was modified by Kakkar et al. method [41]. Each unit of SOD activity was determined as required enzyme concentration for prohibition of creation color at 1 minute, under study conditions. CAT activity was measured by Claiborne method [42] and was based on hydrogen peroxide breakdown. GSH-Px activity was measured by Rotruck et al. method [43] and was expressed as micromole of GSSG/minute/milligram of protein, based on the below reaction:

(1) $$2{\text{H}}_{2}\text{O}+\text{GSSG}\to {\text{H}}_{2}{\text{O}}_{2}+2\text{GSH}$$

### 2.7. Statistical Analysis

The Statistical Package for Social Sciences (SPSS Inc., Chicago, IL, USA), version 13.0, was used for statistical analysis. All data are presented as mean ± SEM. Before statistical analysis, all variables were checked for normality and homogeneity of variance by using the Kolmogorov-Smirnoff and Levene tests, respectively. The data obtained were tested by ANOVA followed by Tukey's post hoc multiple comparison test. *P* < 0.05 was considered statistically significant.

### 2.8. Microscopic Studies

The animals of different groups were sacrificed under light anesthesia (diethyl ether) 1 day after the end of the treatment. A small piece of hepatic tissue from the anterior portion of the left lateral lobe was removed for histological analysis. The sample was fixed by immersing it in 10% neutral-buffered formalin. The sample was then embedded in paraffin, sliced into 5 *μ*m sections, and stained with hematoxylin-eosin for blinded histological assessment. The degree of liver tissue injury was evaluated semiquantitatively according to the method reported by Jamshidzadeh et al. [44]. The stained 5 *μ*m sections were graded as follows: 0, absent; 1, minimal; 2, mild; 3, modest; 4, severe. The histological changes were evaluated in nonconsecutive, randomly chosen ×200 histological fields using light microscope, NIKON ECLIPSE E200.

### 2.9. Statistical Analysis

The Statistical Package for Social Sciences (SPSS Inc., Chicago, IL, USA), version 13.0, was used for statistical analysis. All data are presented as mean ± SEM. Before statistical analysis, all variables were checked for normality and homogeneity of variance by using the Kolmogorov-Smirnoff and Levene tests, respectively. The data obtained were tested by ANOVA followed by Tukey's posthoc multiple comparison test. The Kruskal-Wallis test, followed by Mann-Whitney *U* posttest, was used for the analysis of degree of histopathological liver injury. *P* < 0.05 was considered statistically significant.

## 3. Results

**Figure 1:**
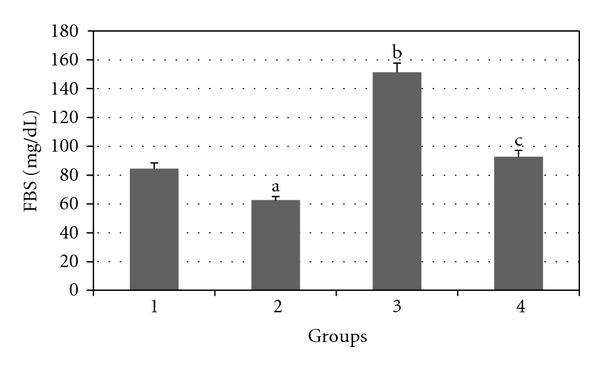
Comparison of the effect of GTE (1.5%, w/v) on blood glucose levels among the experimental groups (mean ± SEM). *P < 0.05, ^a,b^compared to Group 1, ^c^compared to Group 3.

Results of the effect of daily treatment of GTE (1.5%, w/v) for 8 weeks on blood glucose levels of experimental rats are presented in Figure 1. The GTE produced significant hypoglycemic effect in normal (*P* < 0.05) and diabetic (*P* < 0.01) rats after 8 weeks of administration.

**Figure 2:**
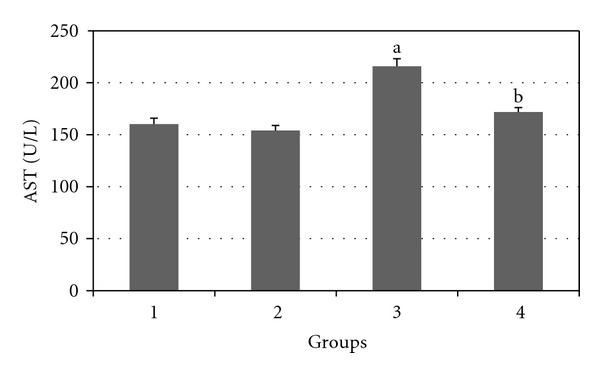
Comparison of the effect of GTE (1.5%, w/v) on serum AST level among the experimental groups (mean ± SEM). *P < 0.05, ^a^compared to Group 1, ^b^compared to Group 3.

**Figure 3:**
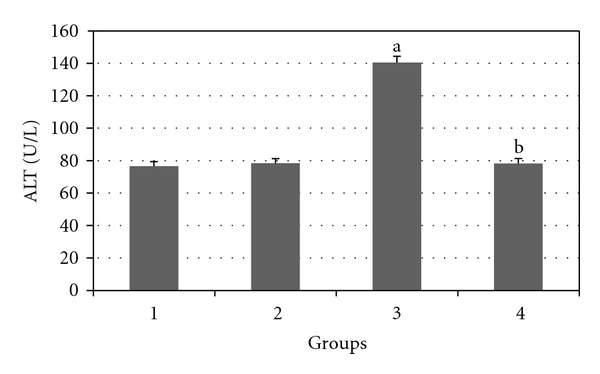
Comparison of the effect of GTE (1.5%, w/v) on serum ALT level among the experimental groups (mean ± SEM). *P < 0.05, ^a^compared to Group 1, ^b^compared to Group 3.

**Figure 4:**
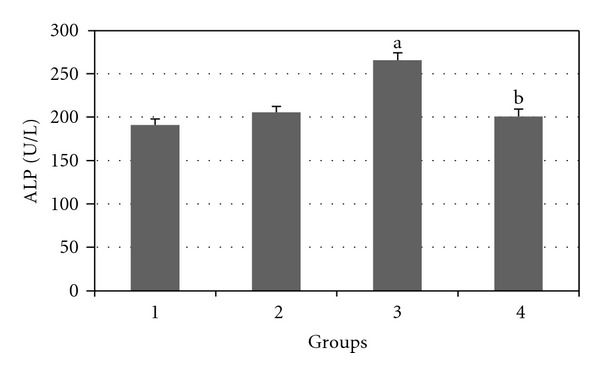
Comparison of the effect of GTE (1.5%, w/v) on serum ALP level among the experimental groups (mean ± SEM). *P < 0.05, ^a^compared to Group 1, ^b^compared to Group 3.

**Figure 5:**
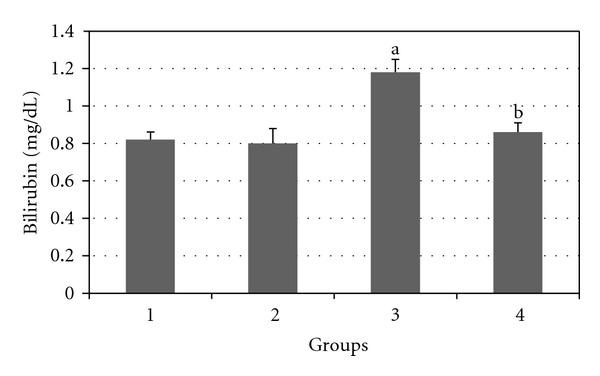
Comparison of the effect of GTE (1.5%, w/v) on serum bilirubin level among the experimental groups (mean ± SEM). *P < 0.05, ^a^compared to Group 1, ^b^compared to Group 3.

**Figure 6:**
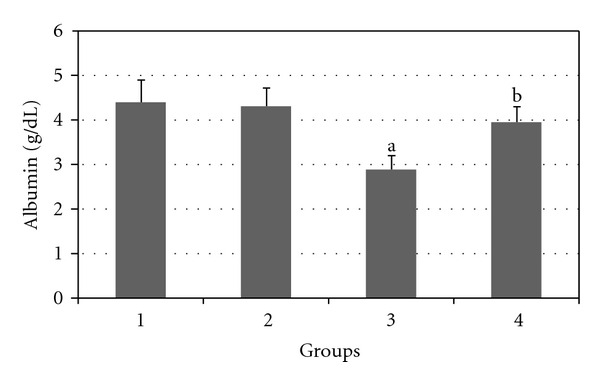
Comparison of the effect of GTE (1.5%, w/v) on serum albumin level among the experimental groups (mean ± SEM). *P < 0.05, ^a^compared to Group 1, ^b^compared to Group 3.

Figures 2, 3, 4, 5, and 6 show the effects of GTE on the serum levels of markers of liver injury (ALT, AST, ALP, and bilirubin) in diabetic rats. ALT, AST, ALP and bilirubin serum contents in Group 3 were found to be significantly increased as compared to Group 1 (*P* < 0.05) and these parameters in Group 4 were significantly decreased as compared to Group 3 (*P* < 0.05). The albumin serum level in Group 3 was significantly decreased as compared to Groups 1 (*P* < 0.05) and this parameter was significantly increased in Group 4 as compared to Group 3 (*P* < 0.05).

**Figure 7:**
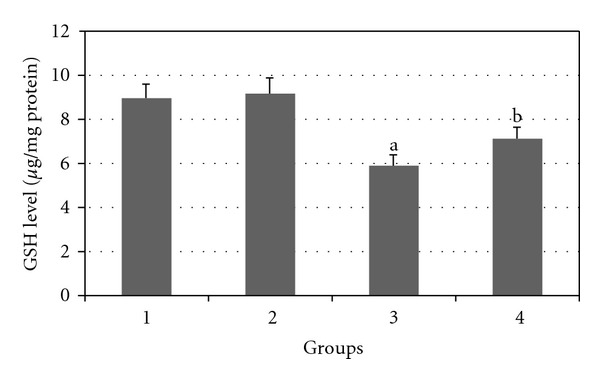
Comparison of the effect of GTE (1.5%, w/v) on liver GSH content among the experimental groups (mean ± SEM). *P < 0.05, ^a^compared to Group 1, ^b^compared to Group 3.

**Figure 8:**
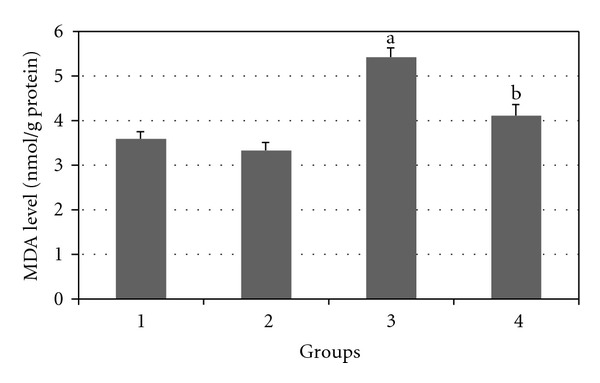
Comparison of the effect of GTE (1.5%, w/v) on liver MDA content among the experimental groups (mean ± SEM). *P < 0.05, ^a^compared to Group 1, ^b^compared to Group 3.

**Figure 9:**
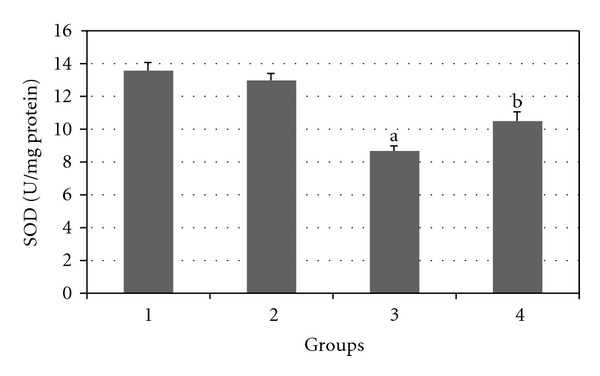
Comparison of the effect of GTE (1.5%, w/v) on liver SOD activity among the experimental groups (mean ± SEM). *P < 0.05, ^a^compared to Group 1, ^b^compared to Group 3.

**Figure 10:**
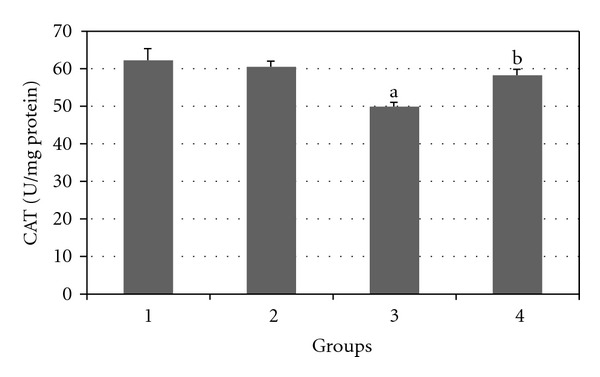
Comparison of the effect of GTE (1.5%, w/v) on liver CAT activity among the experimental groups (mean ± SEM). *P < 0.05, ^a^compared to Group 1, ^b^compared to Group 3.

**Figure 11:**
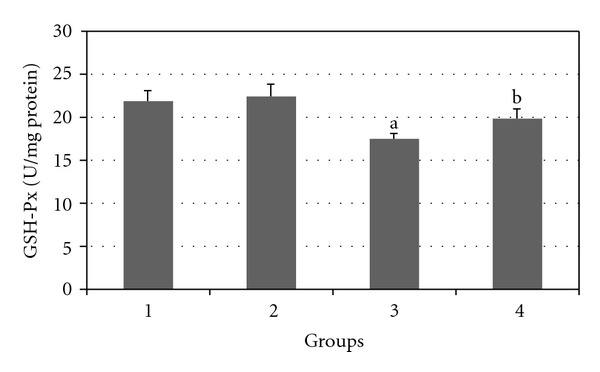
Comparison of the effect of GTE (1.5%, w/v) on liver GSH-Px activity among the experimental groups (mean ± SEM). *P < 0.05, ^a^compared to Group 1, ^b^compared to Group 3.

Figures 7, 8, 9, 10, and 11 show the effects of GTE on antioxidative activity in liver tissue of diabetic rats. MDA contents of the liver tissue in Groups 3 were found to be significantly increased as compared to Group 1 (*P* < 0.05) and liver MDA level in Group 4 was significantly decreased as compared to Group 3 (*P* < 0.05). The GSH, SOD, CAT, and GSH-Px contents of the liver in Group 3 were significantly decreased as compared to Group 1 (*P* < 0.05) and GSH, SOD, CAT, and GSH-Px activity were increased in Group 4 as compared to Group 3 (*P* < 0.05).

**Figure 12:**
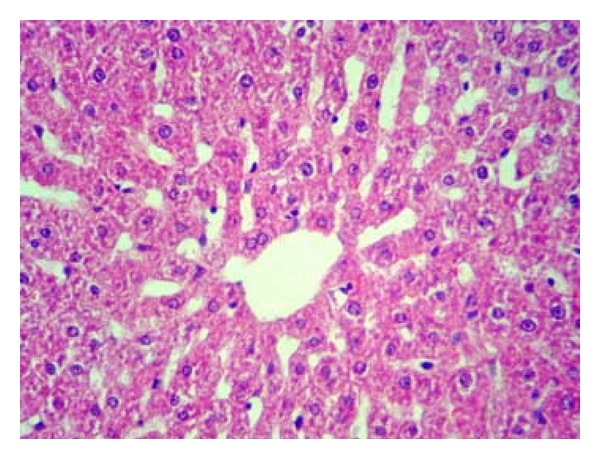
Microscopic appearance from the liver tissue of a rat belonged to healthy control group showing normal hepatic structure (H&E, 100x).

**Figure 13:**
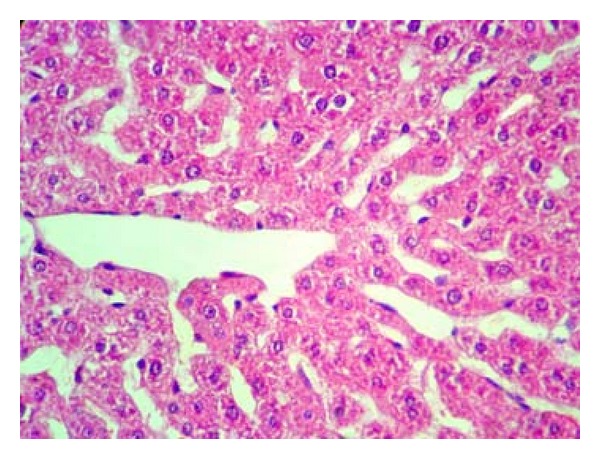
Microscopic view from the liver tissue of a rat belonged to non-diabetic rats + GTE (1.5%, w/v) treatment group. There is no treatment-related lesion in the section (H&E, 100x).

**Figure 14:**
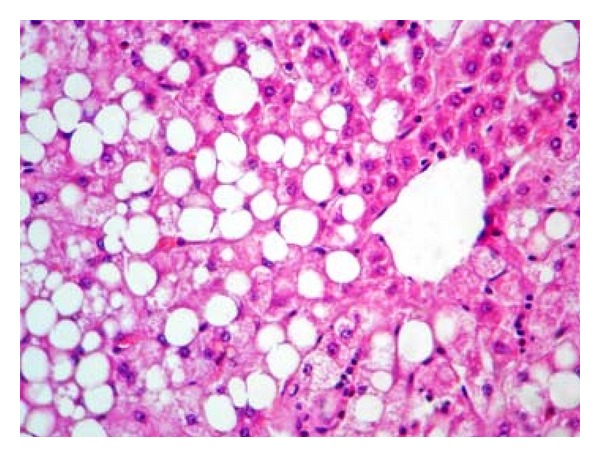
Histologic appearance from the liver tissue of a rat belonged to diabetic group showing macrovesicular fatty change in centrilobular portion (H&E, 100x).

**Figure 15:**
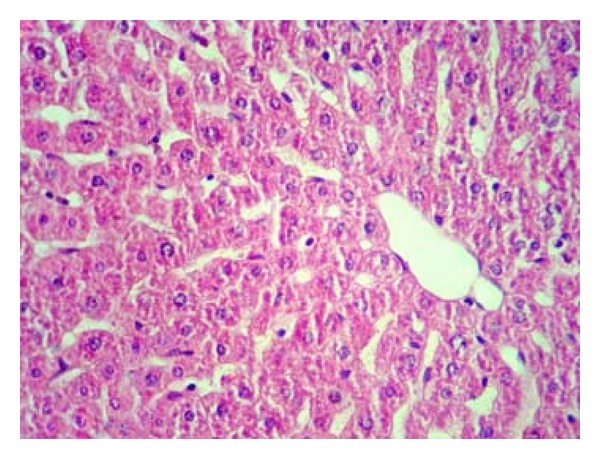
Histologic appearance from the liver tissue of a rat belonged to diabetic rats + GTE (1.5%, w/v) treatment group showing no considerable pathologic change (H&E, 100x).

Pathologically, liver histological structure was normal in healthy control group (Figure 12). In Group 2 also there were no pathological changes so that hepatic lobular structure seemed quite normal (Figure 13). In Group 3, diabetic rats showed fatty changes in centrilobular portions of the livers (Figure 14). Finally, in Group 4, GTE prevented the pathologic changes and no considerable fatty change was observed (Figure 15). Quantitative microscopic results of experimental rats are presented in Table 1.

## 4. Discussion

In current study, oral administration of green tea extract (1.5%, w/v) produced significantly reduction of blood glucose level in healthy normal rats. In addition, green tea extract caused significant hypoglycemic effect in diabetic rats. Such a phenomenon of hypoglycemic response with green tea extract has already been reported [45–47].

In the current study, significant decline in serum albumin level and elevations in markers of liver injury (ALT, AST, ALP, and bilirubin) reflects the hepatocytes injury in experimental diabetes. This result is consistent with the findings reported by Ramesh et al. [48].

Individuals with type 2 diabetes have a higher incidence of liver function test abnormalities than individuals who do not have diabetes [20]. This finding is also in line with our results. The data of our study also revealed that daily treatment of green tea extract markedly improves biochemical and histopathological status of rats with streptozotocin-induced diabetes.

Liver function tests (LFTs) are commonly used in clinical practice to screen for liver disease, monitor the progression of known disease, and monitor the effects of potentially hepatotoxic drugs. The most common LFTs include the serum aminotransferases, alkaline phosphatase, bilirubin, and albumin. Hepatocellular damage causes release of these enzymes into circulation. Increase in serum levels of AST shows hepatic injuries similar to viral hepatitis, infarction, and muscular damages. ALT, which mediates conversion of alanine to pyruvate and glutamate, is specific for liver and is a suitable indicator of hepatic injuries. Increased levels of these enzymes are an indicator of cellular infiltration and functional disturbance of liver cell membranes [49]. In addition, ALP is membrane bound and its alteration is likely to affect the membrane permeability and produce derangement in the transport of metabolites [50]. On the other hand, bilirubin and albumin values are associated with the function of hepatic cells [51].

Return of the above enzymes to normal serum values following green tea extract treatment may be due to prevention of intracellular enzyme leakage resulting from cell membrane stability or cellular regeneration [52]. Effective control of bilirubin and albumin shows early improvement of functional and secretory mechanism of hepatic cells.

In this study, histopathological evaluation of liver tissues showed fatty changes in centrilobular portions of the livers in diabetic rats. These results are in line with the findings reported by Ramesh et al., who observed the hepatoprotective action of Umbelliferone in streptozotocin-induced diabetic rats [48]. With green tea treatment in diabetic rats no considerable fatty change was observed indicating the protective effect of green tea against hepatic complications of diabetes. However, pathologic findings are matched with biochemical results.

In this study, significant (*P* < 0.05) reduction of GSH and antioxidant enzymes (SOD, CAT, and GSH-Px) activity as well as significant (*P* < 0.05) increased lipid peroxidation reflects oxidative stress of the liver in experimental diabetes. These results are in line with the findings reported by Feillet-Coudray et al., who observed that STZ-induced diabetes in rat was accompanied with an increase in the susceptibility to lipid peroxidation [19]. The data of our study also revealed that daily treatment of green tea extract markedly improves antioxidant status of liver tissue of rats with streptozotocin-induced diabetes as GSH level and antioxidant enzymes activities comprising SOD, CAT, and GSH-Px significantly (*P* < 0.05) increased and MDA level markedly (*P* < 0.05) decreased.

GSH (an important part of the nonenzymatic antioxidant system) is a major nonprotein thiol in living organisms, which plays a central role in coordinating the body's antioxidant defense processes. Perturbation of GSH status of a biological system can lead to serious consequences. Elevation in MDA level and reduction in GSH stores of liver tissue of diabetic rats suggest that oxidative stress due to freeradical damage is one of the possible mechanisms in the pathophysiology of diabetic hepatopathy. On administration of Green tea extract, the MDA levels have decreased and the GSH levels have increased. This indicates that in the presence of Green tea extract there is an improvement in the oxidative stress. Increased oxidative stress in the tissues of streptozotocin diabetic rats was similarly reported. This was said to be a contributory factor in the development of the complications of diabetes [53, 54]. It was observed in that study that GSH administration reverses these effects [54]. The data of our study also revealed that daily treatment of Grean tea extract markedly improves antioxidant status of liver tissue of rats with streptozotocin-induced diabetes. Oxidative stress is produced during normal metabolic process in the body as well as induced by a variety of environmental factors and chemicals. Oxidative stress has been shown to have a significant effect in the causation of diabetes as well as diabetes-related complications in human beings [55]. Oxidative stress in diabetes has been shown to coexist with a reduction in the antioxidant status. The exact role of oxidative stress in the etiology of human diabetes is however not known. Oxidative stress has been shown to produce glycation of proteins, inactivation of enzymes, and alterations in structural functions of collagen basement membrane [8]. Oxidative stress may have significant effect in the glucose transport protein (GLUT) or at insulin receptor [56]. Scavengers of oxidative stress may have an effect in reducing the increased serum glucose level in diabetes and may alleviate the diabetes as well as reduce its secondary complications. SOD, CAT, and GPx constitute a mutually supportive team of defense against ROS. SOD is a metalloprotein and is the first enzyme involved in the antioxidant defense by lowering the steady-state level of O_2_^–^. In hyperglycaemia, glucose undergoes autooxidation and produces superoxide and it produces free radicals that in turn lead to lipid peroxidation in lipoproteins. CAT is localized in the peroxisomes or the microperoxisomes, which catalyses the decomposition of H_2_O_2_ to water and oxygen and thus protects the cell from oxidative damage produced by H_2_O_2_. GPx catalyses the reaction of hydroperoxides with reduced glutathione to form glutathione disulphide (GSSG) and the reduction product of the hydroperoxide. In our study, decline in the activities of these enzymes in liver tissue of streptozotocin-induced animals and attainment of near normalcy in green tea extract treated rats indicate oxidative stress elicited in hepatic tissue of diabetic rats had been nullified due to the effect of the extract.

However, evidence suggests that oxidative stress and free radicals play an important role in the pathogenesis of diabetes mellitus and diabetic complications [6]. Liver is one of the most important organs that maintains blood glucose levels within normal limits thus enhancement of blood sugar yield to imbalance of oxidation-reduction reactions in hepatocytes, so that, hyperglycemia through increasing in AGEs (advanced glycation end products) facilities free radicals production via disturbance in ROS production (reactive oxygen species) such as SOD and CAT [57–59]. Hence, it reveals that diabetic hepatic injuries result from several agents and is not controllable only via inhibition of hyperglycemia [60]. Namely, although in early stages of diabetes, tissues injuries are induced via hyperglycemia, their progress in latter stages are not related to hyperglycemia [61]. Therefore, monitoring of blood glucose levels solely is not sufficient in retarding diabetes complications. Thus, a suitable drug must have both antioxidant and blood glucose decreasing properties [62].

Green tea extract contains polyphenols (e.g., catechin, epicatechin, epigallocatechin, and their gallates), tannin, and caffeine. The extract also includes pyrroloquinoline quinone, a newly discovered vitamin [63]. Some constituent components have been shown to enhance the basal and insulin-stimulated glucose uptake of rat adipocytes [28], to inhibit intestinal glucose uptake by inhibiting the sodium-dependent glucose transporter of rabbit intestinal epithelial cells [64], and to reduce serum glucose level in alloxan-diabetic rats [65]. It has been suggested that catechins, antioxidant compounds present in green tea, may improve the defence system of the organism as demonstrated in several in vitro and ex vivo models [66–68]. Green tea extracts are more stable than pure epigallocatechin gallate, the major constituents of green tea, because of the presence of other antioxidant constituents in the extract [69]. In general, herbal medicines are complex mixtures of different compounds that often act in a synergistic fashion and exert their full beneficial effect as total extracts [70]. However, a multitude of herbs, spices, and other plant materials as useful source of natural antioxidants have been described for the treatment of diabetic complications throughout the world. In this manner, Saradha Devi et al. declared that *Cynodon dactylon* has very good antioxidant and hepatic protective effect of normal oxidative stress in Balb/c mice [71]. These similar results were obtained earlier in dry stem crude extraction of *Tinospora cordifolia* and polyphenols extracts in tea [72]. Also, Gohil et al. showed hypoglycaemic and hypolipidemic effects of *Eugenia jambolana* seed and *Aegle marmelos* leaf extracts in alloxan-induced diabetic rats [73].

The results of the present study demonstrate that daily treatment of diabetic rats by green tea extract markedly improves antioxidant status in liver tissue. On the other hand, we found that green tea extract improved serum biomarkers of liver tissue injury and histopathologic properties of this organ. It is therefore likely that green tea extract is prophylactic against diabetic complications and ameliorates diabetic hepatopathy through its antioxidant potential. On the other hand, hyperglycemia is the primary symptom of diabetes and is blamed for the complications of diabetes because elevated glucose concentration directly injures cells and induces lipid peroxidation [74]. Whether this reflects oxidative stress-induced liver injury or direct glycemic injury of liver remains to be determined. Also, information on the occurrence of oxidative stress in the liver tissue at this early stage of diabetes remains undetermined. Taken in all, the use of this plant in diabetes is then supported but the precise active substance(s) of green tea, site(s), and cellular and molecular mechanism(s) of its pharmacological effect are still to be determined. In addition, the possible long-term toxic effects of green tea extract and protective effects of different doses of that also remain to be clarified.

## 5. Conclusion

This study demonstrated green tea extract has hepatoprotective activity in streptozotocin-induced diabetic rats. Many question related to antioxidant effect of Green tea extract remain unanswered. Much more work is clearly needed before phytotherapy for diabetic hepatopathy can be advanced to clinic.

## Figures and Tables

**Table 1:** Effect of GTE (1.5%, w/v) on hepatic injuries of diabetic rats (mean ± SEM).

Groups	Treatment	Degree of liver tissue injury	The Kruskal-Wallis test
1	Healthy control rats	0.0 ± 0.0	
2	Nondiabetic rats + GTE (1.5%, w/v) treatment	0.0 ± 0.0	*P* < 0.001
3	Diabetic rats	2.80 ± 0.65^a^	
4	Diabetic rats + GTE (1.5%, w/v) treatment	0.85 ± 0.17^b^	

0 = without injury, 1 = minimum injury, 2 = mild injury, 3 = moderate injury, 4 = sever injury (*n* = 10)  ^a^compared to Group 1, ^b^compared to Group 3.
